# Coupled Effects of Temperature and Humidity on Fracture Toughness of Al–Mg–Si–Mn Alloy

**DOI:** 10.3390/ma16114066

**Published:** 2023-05-30

**Authors:** Ibrahim Alqahtani, Andrew Starr, Muhammad Khan

**Affiliations:** Centre for Life-Cycle Engineering and Management, School of Aerospace, Transport and Manufacturing, Cranfield University, College Road, Cranfield MK43 0AL, UK

**Keywords:** Al–Mg–Si–Mn alloy, fracture toughness, coastal environments, polynomial model, failure mechanism

## Abstract

The combined effect of temperature and humidity on the fracture toughness of aluminium alloys has not been extensively studied, and little attention has been paid due to its complexity, understanding of its behaviour, and difficulty in predicting the effect of the combined factors. Therefore, the present study aims to address this knowledge gap and improve the understanding of the interdependencies between the coupled effects of temperature and humidity on the fracture toughness of Al–Mg–Si–Mn alloy, which can have practical implications for the selection and design of materials in coastal environments. Fracture toughness experiments were carried out by simulating the coastal environments, such as localised corrosion, temperature, and humidity, using compact tension specimens. The fracture toughness increased with varying temperatures from 20 to 80 °C and decreased with variable humidity levels between 40% and 90%, revealing Al–Mg–Si–Mn alloy is susceptible to corrosive environments. Using a curve-fitting approach that mapped the micrographs to temperature and humidity conditions, an empirical model was developed, which revealed that the interaction between temperature and humidity was complex and followed a nonlinear interaction supported by microstructure images of SEM and collected empirical data.

## 1. Introduction

Al–Mg–Si alloys are commonly used in various structural applications like aircraft wings, rotor blades [1], and marine facilities [2,3,4] due to their superior specific strength compared to other aluminium alloys in the 6xxx series. These alloys offer several benefits, such as excellent strength-to-weight ratio, sufficient strength, and high corrosion resistance [5]. In engineering, fracture toughness values are often utilised to characterise materials, evaluate their performance, and ensure their quality in typical structural applications. The coastal environmental factors such as temperature, humidity, and corrosion also impact the fracture toughness of the material [6]. Zakaria [7] studied the effect of increasing temperature up to 75 °C and exposure time up to 5 days in a 3.5% NaCl solution on aluminium alloy. The results conclude that increased temperature leads to a higher corrosion rate. When aluminium is placed in a 3.5% NaCl solution (pH-6.1), it can undergo corrosion due to chloride ions [8]. The corrosion process can lead to aluminium oxide and hydrogen gas formation. The rate and extent of corrosion depend on several factors, such as the surface area, temperature, pH, and concentration of the NaCl solution [9].

The ability of aluminium to resist corrosion is linked to the durability of the naturally formed oxide layer that protects its surface. However, aluminium is prone to pitting corrosion in a higher pH range (8.1 for marine water) at room temperature and 80 °C [10]. Although oxygen may cause localised corrosion, its impact is relatively limited. The formation of oxide layers such as FeO, Al_2_O_3_, CuO, ZnO, etc., is observed in marine water above 30–80 °C [11]. Above 70 °C and up to 150°C, the propensity of pitting corrosion progressively disappears due to the formation of an aluminium oxide layer [12]. Corrosion caused by atmospheric conditions occurs when the relative humidity in the atmosphere surpasses the equilibrium relative humidity over any saturated solution on the surface of the metal. This typically occurs in humid environments [13].

Moreover, temperature can potentially raise the salinity levels of seawater and consequently increase its aggressiveness due to evaporation [14]. The evaporation of harmful airborne substances, such as chlorides, can affect the structures [15]. On the other side, when chloride penetration occurs at a faster pace and greater depth, it can result in microcracks within the material. Additionally, the relative humidity level in the marine environment surrounding the material can also play a role in sustaining various chemical deterioration processes.

The weather data show that the temperature in Middle Eastern regions can reach nearly 60 °C during the summer months [15]. High ambient temperatures characterise the Gulf region from 13 to 60 °C with salt-laden humidity (40% to 95%) [14,15] with a chloride ion concentration of about 3.6%. Environmental factors were responsible for 52% of the failures in aircraft components during service, according to a report from the aviation system [16,17,18,19]. The operation conditions of these aircraft components in the coastal areas were affected by the temperature, humidity, and corrosive environment. When the heat generated by aircraft equipment during operation is considered, it can result in extreme temperatures that may exceed 70 °C, which can impact the performance of components [1,11]. These exposure conditions can cause the temperature of structures to rise to 80 °C, and the humidity can reach 90% [1]. 

Thus, a comprehensive approach is required to relate the interdependency of humidity and temperature with Al–Mg–Si–Mn alloy properties in a corrosive environment. However, there are few studies on the environmental failure behaviour of aluminium alloys, especially cracking failure recurrence behaviour under varying temperature or humidity conditions, but not combinedly. Studying the combined effect of temperature and humidity on the material’s fracture toughness is essential in this connection. 

The present study aims to investigate the fracture toughness of the Al–Mg–Si–Mn alloy under the specific conditions of a coastal environment, considering the influence of varying temperatures and humidity. The novelty of this work lies in three key aspects. First, it involves a thorough investigation of fracture toughness in a coastal environment, which is crucial due to the unique challenges caused by high humidity and potential exposure to corrosive elements. Second, an empirical model has been developed to accurately predict the fracture toughness values, offering valuable insights for engineering applications and material design in coastal environments. Lastly, a novel methodology has been adopted to analyse the nonlinear variation resulting from the combined effect of temperature and humidity by mapping micrographs with the obtained curve. This comprehensive analysis method provides a deeper understanding of the intricate interactions and nonlinear behaviour exhibited by the Al–Mg–Si–Mn alloy in response to changing environmental conditions.

## 2. Experimental Methods

### 2.1. Material 

Aluminium 6082, called Al–Mg–Si–Mn alloy, is frequently used in structural applications [20]. Referring to the information provided in Refs. [5,20], this specific alloy displays excellent strength, weldability, and resistance to wear and corrosion owing to its unique chemical composition. Al–Mg–Si–Mn was supplied by Doré Metal Services Southern Ltd., Kent, UK. The main elements present in it are mentioned in Table 1. 

The T651 state, which prevents elastic recovery after processing, is attained using solution treatment, stress relief by stretching, and artificial ageing at a temperature of about 180 °C. The properties of Al–Mg–Si–Mn alloy in the T651 condition were density = 2.71 g/cc, tensile strength = 348 MPa, yield strength = 320 MPa, elastic modulus = 70 GPa, elongation = 17.5% [21]. Due to its main constituent elements such as Mg, Si, and Mn, they are distinguished by high fracture toughness [22]. Its melting point is 555 °C, thermal conductivity is 180 W/m.K, and thermal expansion is 24 × 10^−6^/K.

### 2.2. Experimentation

**Figure 1:**
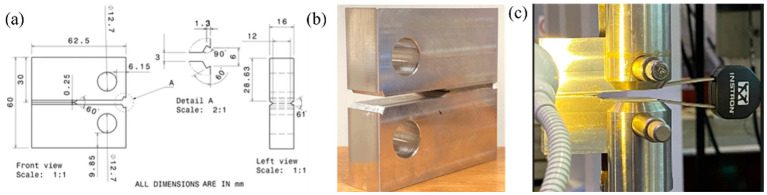
(**a**) The geometry (unit: mm), (**b**) prepared C(T) specimen, and (**c**) experimental setup.

Al–Mg–Si–Mn alloy sheet 16 mm thick was machined to prepare the compact tension (C(T)) specimen as per the required geometry, as illustrated in Figure 1. Out of many standard specimens, C(T) specimens are widely used by many researchers for their simplicity in structure, ease of preparation, testing setups, and fixtures being easily available. Wire cut electrical discharge machining (EDM) was used to prepare the C(T) specimen. The specimens with side grooves cut with a triangle profile [23] have been considered with an opening angle of 61°. The notch introduced using wire-cut EDM may not simulate the crack in the material. The wire diameter in EDM is 0.2 mm width. Thus, the need of introduction of microcrack in the specimen for fracture toughness test. The C(T) specimens were pre-cracked using a servo-hydraulic testing apparatus. Furthermore, a fatigue crack is introduced to the end of the notch for all C(T) specimens while maintaining crack length-to-width (a/W) ratios of 0.54 [24] and fatigue stress ratio of 0.1. Introducing a fatigue crack before conducting the fracture toughness test ensures that the test focuses specifically on the material’s ability to resist crack propagation and fracture rather than just its strength or ductility. This is important because each material has a crack, which is very tiny and sharp [25]. 

The pre-cracked C(T) specimens were immersed in a 3.5 weight per cent NaCl solution at room temperature for 7 days [7] for localised corrosion tests. The samples were taken out after 7 days and allowed to air dry. The thermal chamber was used to simulate the harsh coastal environmental conditions such as temperature and humidity. All the corroded C(T) specimens were kept in the thermal chamber for temperatures 20, 40, 60, and 80 °C and humidity of 40%, 50%, 60%, 70%, 80%, and 90%.

In the coastal environment of the Middle East, the temperature can rise as high as 60 °C, while the operating temperature of machine components may reach up to 70 °C. Therefore, a temperature range of 20 °C to 80 °C was selected to encompass these conditions. Additionally, the humidity in the Middle East region can vary between 40% and 90%.

On a servo-hydraulic testing machine with a load capacity of 100 KN, fracture toughness investigations on C(T) specimens were carried out by maintaining a frequency of 3 Hz. Figure 1b shows the prepared sample, and Figure 1c shows the C(T) specimen in the experimental setup. A 0.1 mm/min displacement rate was maintained during fracture toughness testing. The extensometer/strain gauge was positioned at the knife edge for C(T) specimens. The fracture toughness was determined using the load versus extensometer displacement curves data [24]. Three specimens were tested for each condition, and the average fracture toughness value was considered. Using three specimens for fracture toughness testing is a common practice to validate the results obtained from each specimen. In this study, the objective was to investigate the fracture toughness of Al–Mg–Si–Mn alloy and ensure the validity of the results by using three specimens for testing. 

## 3. Results and Discussion

### 3.1. Subsection Load vs. Crack Opening Displacement

**Figure 2:**
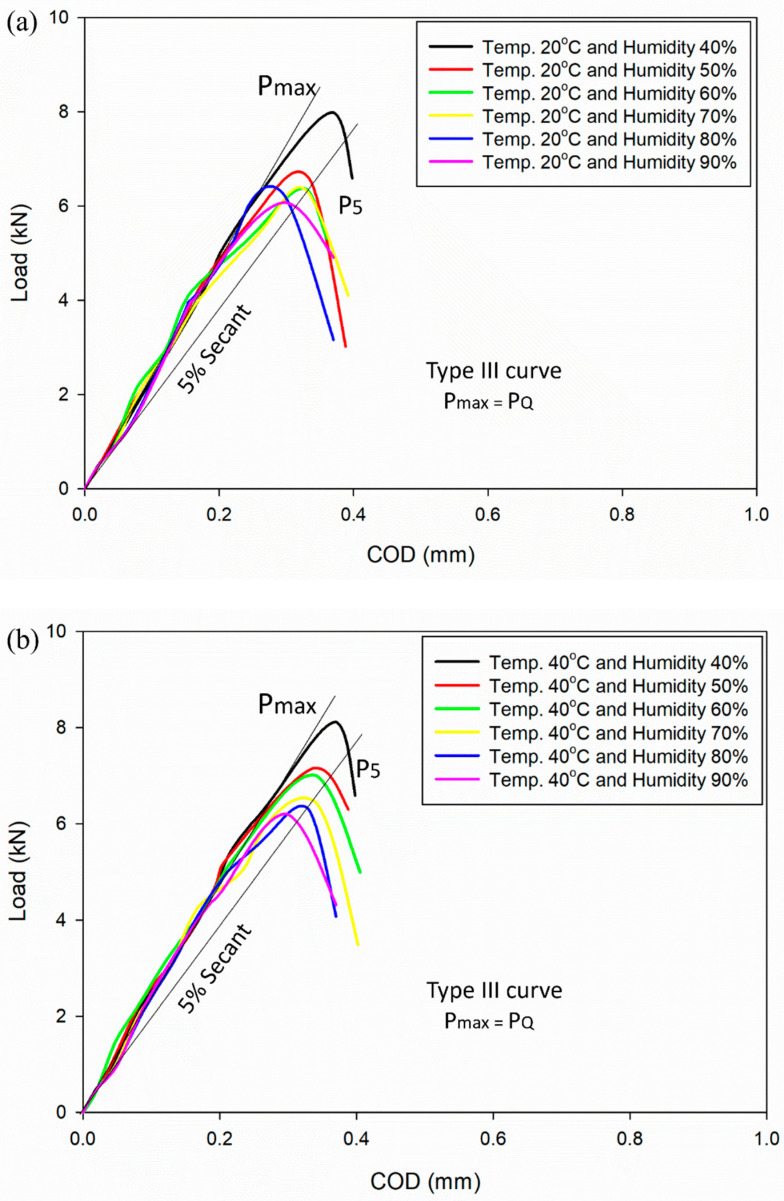

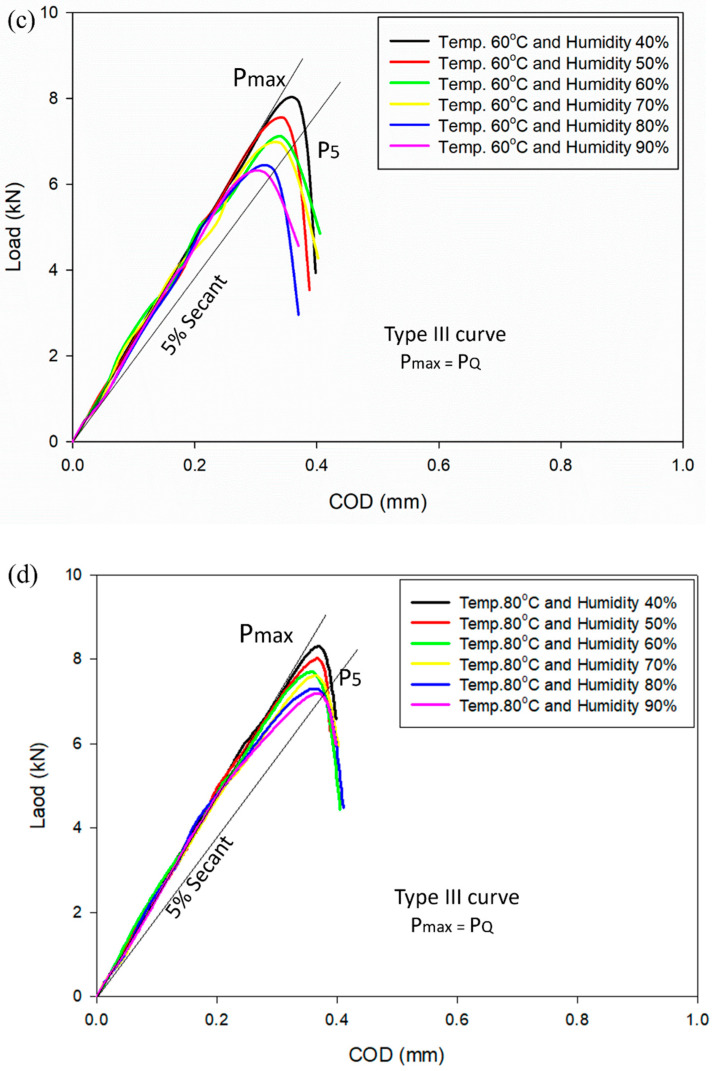
Load vs. COD graph of Al–Mg–Si–Mn alloy with different humidities (**a**) 20 °C, (**b**) 40 °C, (**c**) 60 °C, (**d**) 80 °C.

From the fracture toughness testing, the load applied and strain gauge readings were recorded and analysed by plotting the load versus COD plot. Figure 2a–d displays the load versus COD of an Al–Mg–Si–Mn alloy under varied temperature and humidity conditions. 

Figure 2a–d depicts the determination of the critical load (*P_Q_*) value using curve fitting techniques, specifically by drawing a 5% secant line to the maximum load (*P*_max_) on the experimental data. The plots show that all load versus COD curve cases match the type III curve [24,26], which indicates *P*_max_ = *P_Q_*. Moreover, P_5_ as shown in Figure 2 is the load determined using the 5% secant method.

From plots of load vs. COD, it can be observed that as the humidity increases, the load-carrying capacity of an Al–Mg–Si–Mn alloy decreases. A humidity level of 40% at a temperature of 20–80 °C typically does not significantly impact the load-carrying ability of aluminium, as aluminium is generally not susceptible to moisture absorption or degradation [27]. From Figure 2d, it can be observed that the temperature of 80 °C and humidity level of less than 50% have very little impact on the load-carrying capacity of Al–Mg–Si–Mn alloy. However, exposure to high humidity levels, 50–90% and at temperature (20–80 °C) can lead to aluminium surface corrosion, weakening the material and reducing its load-carrying capacity [28,29].

### 3.2. Fracture Toughness

Using Equation (1) [24], conditional fracture toughness *K_Q_* is calculated from each test’s *P_Q_* value and the measured crack length using Equation (1). Along with Equations (2) and (3), the *P*_max_/*P_Q_* ratio satisfies the normative criteria for obtaining the linear elastic plane strain fracture toughness. The primary result of the experimental tests is load-displacement curves.

(1) $$K_{Q}=\frac{P_{Q}}{\sqrt{BB_{N}}\sqrt{W}}f\left(\frac{a}{W}\right)$$

where for CT Specimens,

faW=2+aW1−aW32[0.886+4.64aW−13.32aW2+14.72aW3−5.6aW4]

The primary determinant of fracture toughness is specimen thickness (*B*). When the thickness of the specimen reaches the significant limit, the fracture toughness value seems fairly steady. It is referred to as plane strain fracture toughness, represented by *K*_*Ic*_. In Equations (2) and (3) [26], the following conditions are specified for the plane strain fracture toughness:

(2) $$a,B\geq 2.5{\left(\frac{K_{Q}}{\sigma_{y}}\right)}^{2}$$

(3) $$W\geq 5.0{\left(\frac{K_{Q}}{\sigma_{y}}\right)}^{2}$$

where $\sigma_{y}$ is the yield strength of the material, *B_N_* is the thickness of the specimen at notch, *B* is the thickness of the specimen. 

The dimensions considered here fulfil the conditions of plane strain fracture toughness. As per Equations (2) and (3), the crack length (*a*) and thickness (*B*) of the specimen must be greater than 12.9 mm and width of the specimen (*W*) must be greater than 26 mm for the provisional fracture toughness (*K_Q_*) obtained. However, specimens considered for the present work have *a* = 27 mm and *W* = 50 mm. The fracture toughness of the Al–Mg–Si–Mn alloy for various temperature and humidity conditions is calculated using Equation (1).

**Figure 3:**
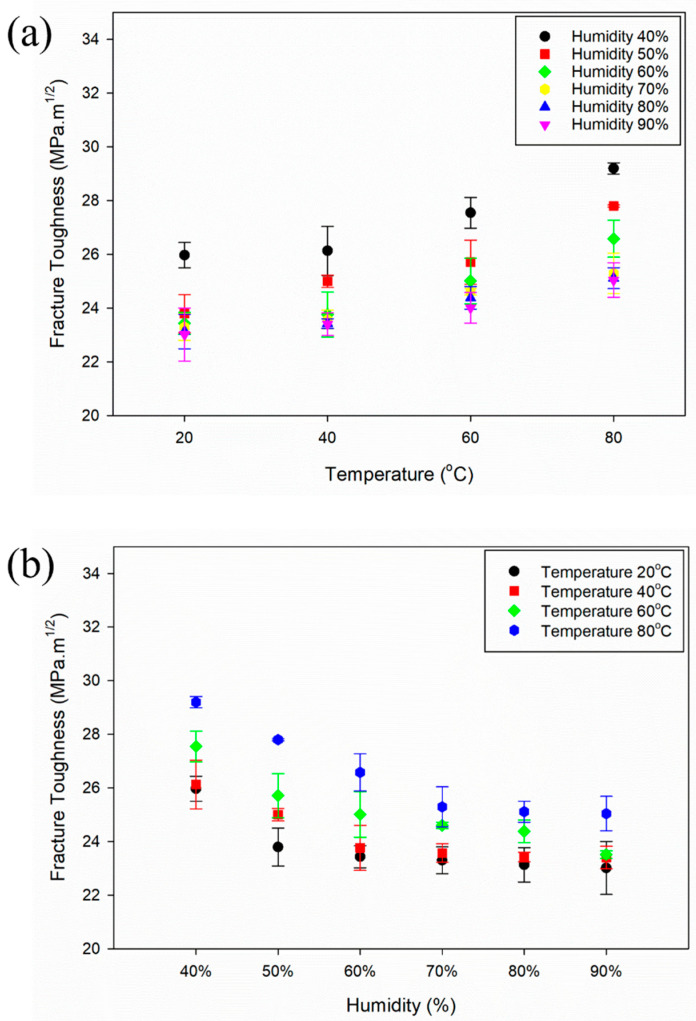
Fracture toughness versus (**a**) temperature and (**b**) humidity conditions.

Figure 3a,b shows the effect of temperature and humidity on the fracture toughness of the Al–Mg–Si–Mn alloy. Figure 3a shows that as the temperature increases, the fracture toughness of the Al–Mg–Si–Mn alloy increases. Figure 3b shows that as the humidity increases, the fracture toughness of the Al–Mg–Si–Mn alloy decreases. The hydrogen embrittlement sensitivity (HES) significantly increases when the humidity increases from 40% to 60% compared to other humidity transitions [28]. The significant increase in HES is thought to be a large contributor to the reduction in the Al–Mg–Si–Mn alloy’s load-carrying capacity and fracture toughness. Hence, the highest reduction in load-carrying capacity was generally observed between 40% and 60% humidity levels at all temperatures (20 °C to 80 °C). Therefore, the Al–Mg–Si–Mn alloy’s load-carrying capacity and fracture toughness is reduced by 10%; as a result, shown in Figure 3. A temperature of 20 °C and high humidity levels (60–90%) have less impact, less than 3%, on the fracture toughness of the Al–Mg–Si–Mn alloy. With the higher humidity levels (70–90%) and at all temperatures (20 °C to 80 °C), the fracture toughness change of the Al–Mg–Si–Mn alloy is nearly identical. The oxide layer forms on the aluminium surface due to exposure to a 3.5 wt. % NaCl acts as a barrier to further corrosion, potentially reducing surface damage and increasing the material’s resistance to corrosion-related cracking [12].

In humid environments, the corrosion rate on the aluminium surface increased as the Al–Mg–Si–Mn alloy corroded. Its reactions led to hydrogen absorption into an aluminium alloy, increasing the voids around the crack tip [28,30]. In this void region, hydrogen diffusion occurs in the Al–Mg–Si–Mn alloy and accumulates at defects such as voids or dislocations. This accumulation of hydrogen causes localised stress concentrations that reduce the material’s ductility and promote the formation of cracks. Further accumulation of hydrogen at the crack tip promotes crack growth. It causes the Al–Mg–Si–Mn alloy to become more brittle, making it more susceptible to fracture under repeated stress [31]. Thus, the Al–Mg–Si–Mn alloy’s load-carrying capacity decreases, reducing the fracture toughness. 

### 3.3. Fracture Surface Morphology

**Figure 4:**
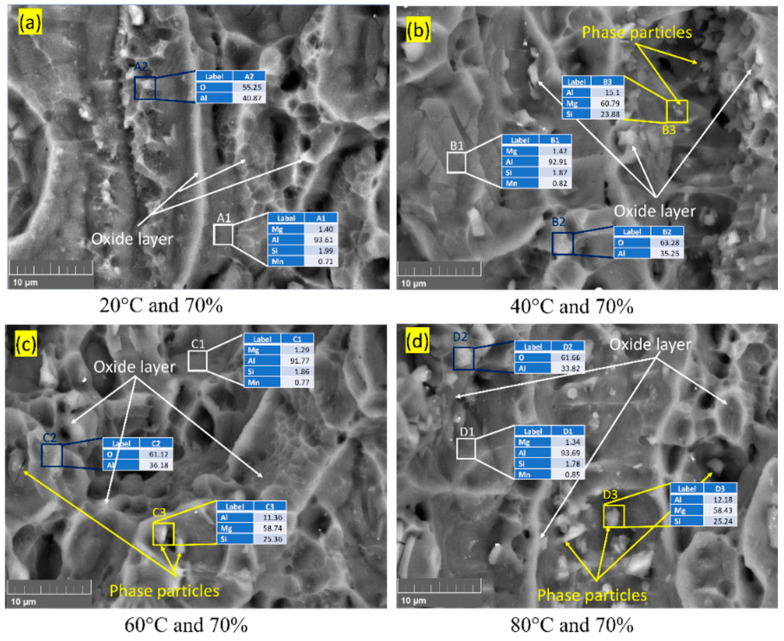
BSE images of Al–Mg–Si–Mn alloy for varying temperature and humidity 70%.

In the Al–Mg–Si–Mn alloy’s fractured surface, Figure 4, the presence of a bright area under backscattered electrons (BSE) imaging indicates an oxide layer’s presence [32], and the Al–Mg–Si–Mn alloy without oxide layer appeared darker or grey [11]. This oxide layer can act as a protective barrier for the Al–Mg–Si–Mn alloy, preventing further corrosion and degradation [33]. 

Due to the increase in temperature, from 20 °C to 80 °C, the percentage elongation of the aluminium alloy increases [34]. Thus, the increased temperature increases the ductility, increasing the material’s fracture toughness [35]. The phase particles were present in the samples tested at 60 °C and 80 °C, significantly less at 40 °C, and no sign in 20 °C temperatures (Figure 4a–d). Thus, it is evident that at temperatures above 60 °C, phase particles are formed. Heating above 70 °C and during the cooling stage, elements such as Mg in the Al–Mg–Si–Mn alloy will begin to cluster together to form small and stable particles called precipitates such as Mg_2_Si intermetallic [36]. These precipitates act as obstacles to dislocation motion and improve the strength of the Al–Mg–Si–Mn alloy. As per Yan Lu et al. [37], average dislocation motion is much less at room temperature, rapidly increasing from 30 °C to 60 °C and slowly after 60 °C, therefore this assured that the increment in temperature from (60 °C to 80 °C), at constant humidity have little impact on the fracture toughness of the Al–Mg–Si–Mn alloy. Since the dislocation motion is significantly less after 60 °C, the formed phase particles act more as a barricade and regulate void formation (shown in Figure 4). Hence, the phase particles reduce the crack nucleation at the crack tip and slow down the crack propagation thus increases fracture toughness. 

**Figure 5:**
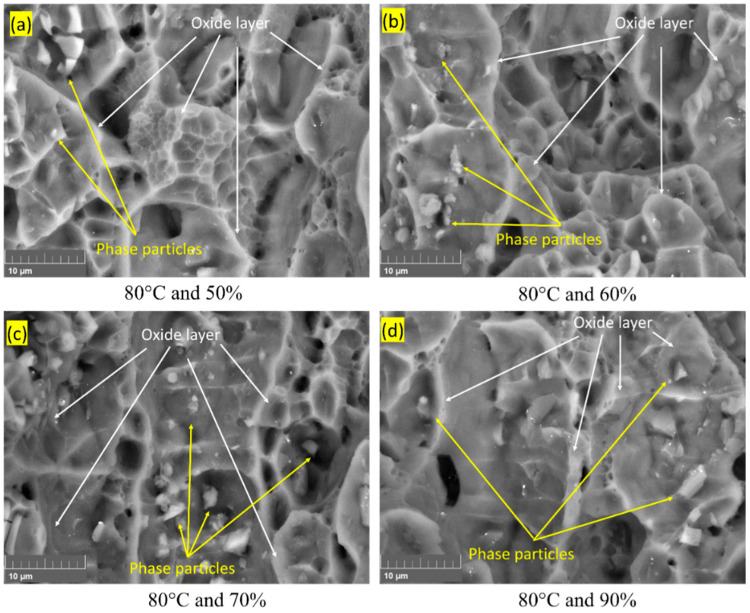
BSE images of Al–Mg–Si–Mn alloy for temperature 80 °C and varying humidity.

From Figure 5, it can be observed that as the increment in humidity at 80 °C, the formation of the phase particles increases. An increase in humidity from 50% to 90% indirectly affects a material’s properties by promoting corrosion or degradation, which leads to changes in the surface morphology and composition of the material’s surface. This corrosion affects the stability and distribution of phase particles. This random distribution of the phase particles, shown in Figure 5, acts as a stress concentrator and creates regions of high stress around the particles. Thus, the formation of crack encounters in these regions will likely propagate and cause a reduction in fracture toughness. 

**Figure 6:**
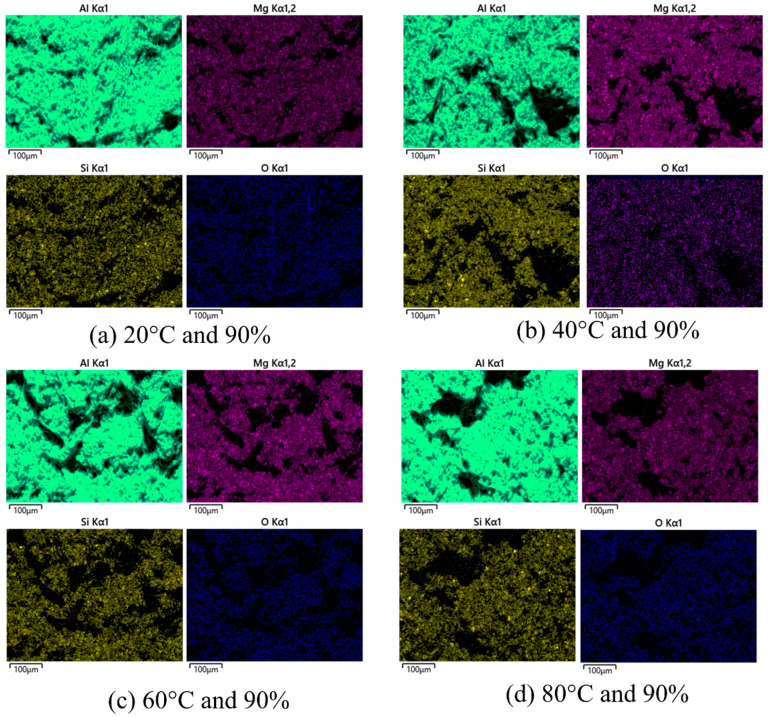
EDS mapping of the fractured surface of the Al–Mg–Si–Mn alloy at different temperatures and at high humidity.

Further analysis, such as energy dispersive spectroscopy (EDS), is assumed to be necessary to confirm the composition of the bright area and the presence of phase particles. Figure 6 shows the EDS mapping of the fractured surface of the Al–Mg–Si–Mn alloy at various temperature and humidity conditions. 

The EDS mapping shows that as the temperature increases from 20 °C to 80 °C, the contents of Mg and Si improve [36,38]. This enhances the strength of the Al–Mg–Si–Mn alloy, thus increasing the load-bearing capacity and, in turn, increasing the fracture toughness. The increment temperature, at higher humidity, increases the corrosion [5], which produces large intermetallic particles and removes the protective oxide layer [4] on the Al–Mg–Si–Mn alloy surface, thus reducing the fracture toughness. 

**Figure 7:**
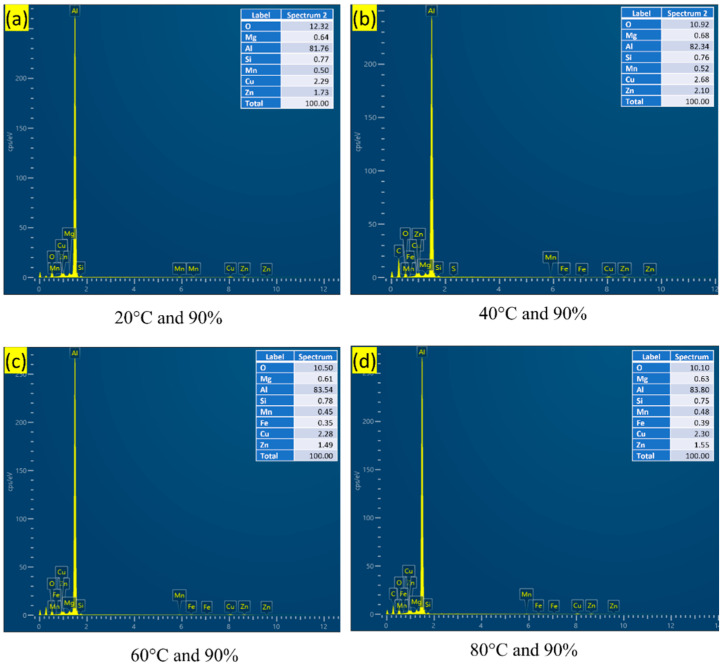
EDS composition of corrosion products of the Al–Mg–Si–Mn alloy at different temperatures and high humidity.

Apart from Al, Si, Mg, and O, the EDS examination of the particles shown in Figure 6 revealed that those phases typically have a significant amount of Cu and Zn shown in Figure 7. Upon exposure to the NaCl solution and atmosphere, i.e., temperature and humidity, Al–Mg–Si–Mn alloy surfaces degrade with the formation of a large variety of corrosion products [39]. The corrosion products observed at 90% humidity were much bulkier than those at 70% and had lesser humidity.

When Al–Mg–Si–Mn alloy is subjected to atmospheric corrosion, it undergoes a complex series of chemical reactions that can form various corrosion products, as shown in Figure 7. Cu and Zn are more anodic (more easily corroded) than aluminium [40,41], so when exposed to a corrosive environment, they will be preferentially corroded over the Al–Mg–Si–Mn alloy. As they dissolve, they can react with other environmental varieties to form corrosion products that can be deposited on the fractured surface of the Al–Mg–Si–Mn alloy. At high humidity conditions, the corrosion of Al–Mg–Si–Mn alloy increases, which reduces the oxide content as indicated by Figure 7a–d. 

**Figure 8:**
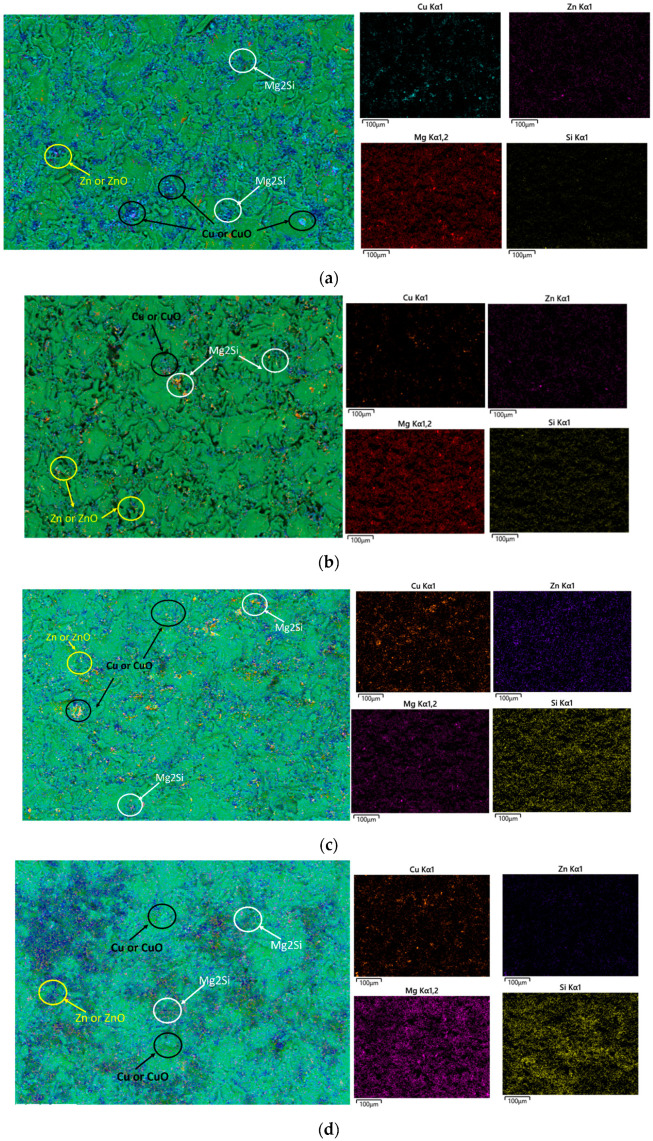
EDS mapping of corrosion products on the fractured surface of the Al–Mg–Si–Mn alloy. (**a**) at 20 °C temperature and humidity 90%. (**b**) at 40 °C temperature and humidity 90%. (**c**) at 60 °C temperature and humidity 90%. (**d**) at 80 °C temperature and humidity 90%.

**Figure 9:**
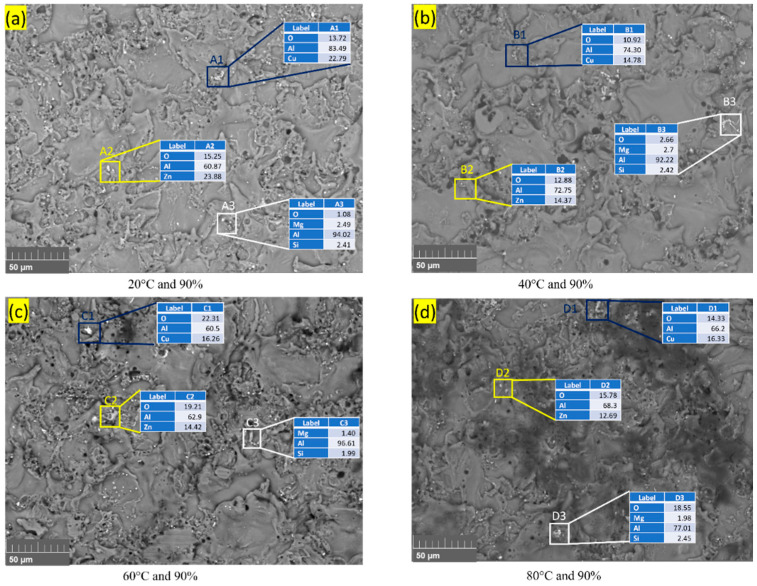
EDS of corrosion products on the fractured surface of the Al–Mg–Si–Mn alloy.

When Al–Mg–Si–Mn alloy is exposed to an increasing temperature at high humidity, the protective oxide layer on the surface of the alloy can break down, leading to increased corrosion rates. As a result, corrosion products might form on the surface of the alloy, which can include metal ions such as copper Cu and Zn and Mg_2_Si [41], as shown in the EDS plot Figure 8a–d and Figure 9a–d. These corrosion products form a protective layer on the surface of the Al–Mg–Si–Mn alloy. This layer helps slow down the corrosion of the Al–Mg–Si–Mn alloy by acting as a barrier to the corrosive environment. Thus, the increased load-carrying capacity and fracture toughness increases as temperature increases.

**Figure 10:**
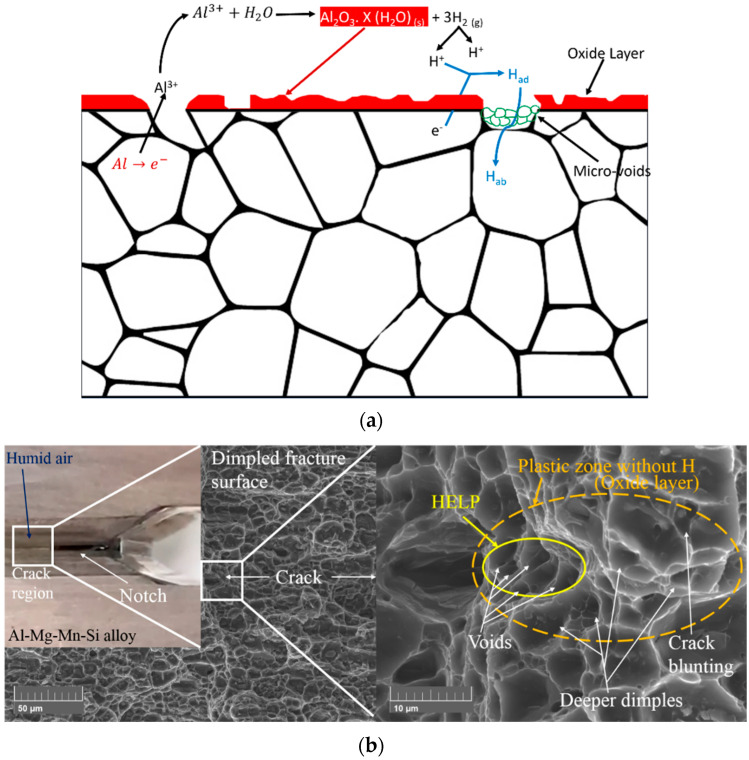
(**a**). The hydrogen absorption mechanism in Al–Mg–Si–Mn alloy. (**b**). SE images of hydrogen-enhanced localised plasticity (HELP) mechanism in Al–Mg–Si–Mn alloy at temperature 20 °C and 90% humidity.

Al–Mg–Si–Mn alloy-subjected corrosion reactions in humidity environments result in hydrogen absorption into aluminium alloy during deformation. The deformation primarily occurs in the region where the hydrogen concentration is critical. The metal lattice fractures once the critical deformation is attained in the particular localised zone placed ahead of the crack tip [31]. When specimens are subjected to atmospheres with high humidity, it has been reported that high-fugacity hydrogen gas produced by the reaction of aluminium with water vapor inhibits the development of environmentally assisted cracks through hydrogen embrittlement [28]. Figure 10a illustrates the hydrogen absorption mechanism of Al–Mg–Si–Mn alloy during deformation, whereby dislocation motion causes hydrogen absorption into the surface of the aluminium alloy in a humid environment.

The reduction in water at the alloy surface can produce hydrogen atoms that have the potential to be absorbed into the alloy and then recombine to form H_2_ gas, as shown in Equation (4) presented below [42];

2Al (s) + (3 + X) H_2_O (g) → 3H_2_ (g) + Al_2_O_3_. X (H_2_O) (s) (4)

where X is the degree of hydration.

Figure 10b shows the Al–Mg–Si–Mn alloy’s hydrogen-enhanced localised plasticity (HELP) mechanism in a humid environment. There is a chance that two plastic zones, the high H concentration zone (HELP) and oxide layer (Plastic zone without H) zone [31], would occur at the end of the crack region due to hydrogen embrittlement on the Al–Mg–Si–Mn alloy’s fractured surfaces. Due to the high hydrogen content, the HELP zone has many voids, which could lead to crack nucleation. The surrounded oxide zone has crack blunting, deeper dimples, and higher overall strain due to micro-void coalescence [30]. A dislocation moving through a metal creates a localised deformation in the crystal lattice [24,37]. This deformation attracts the hydrogen atoms and gets trapped in the dislocation region. Thus, the formation of hydrogen-rich regions near the dislocation might cause embrittlement in the Al–Mg–Si–Mn alloy. The crack nucleation causes early crack propagation and reduces the threshold fracture toughness of the material. Due to this, the plane strain fracture toughness of the Al–Mg–Si–Mn alloy decreases with the increase in high humidity conditions.

## 4. Validation of Results

### 4.1. Empirical Modelling

In general, the effect of humidity and temperature on Al–Mg–Si–Mn alloy can lead to complex changes in its properties, and the resulting curves may not necessarily be linear. Al–Mg–Si–Mn alloy is susceptible to corrosion when exposed to high humidity and certain temperatures. The degree and rate of corrosion can depend on various factors, including the humidity level, temperature, presence of other chemicals, and duration of exposure. However, experiments carried out by various researchers describe that the effect of humidity [28] and temperature [34,35,40] on the mechanical properties of aluminium alloy is a function of nonlinearity. 

The fracture toughness experiments were carried out for various couple loads such as temperature 20 to 80 °C and humidity 40 to 90% on Al–Mg–Si–Mn alloy. The results obtained emphasise the possibility that humidity and temperature variations may have contributed to the scatter of results. For these scattered points, it is required to fit the curve to find a mathematical function that best approximates a set of data points. This also helps to identify trends, make predictions, and provide insight into the underlying mechanisms of fracture toughness under coastal region temperature and humidity conditions. 

In MATLAB, polynomial functions of two variables were employed to model the complex relationships between temperature and humidity on fracture toughness. Using these polynomial functions, coefficients were assigned by the weights to determine the shape of the surface [43]. Curve-fitting techniques were employed to model the surface’s shape [44]. Specifically, the cubic polynomial functions were used to adjust the coefficients and achieve the best fit for the given data. The resulting model might accurately capture the relationship between fracture toughness, temperature, and humidity. The resulting polynomial equation is mentioned in Equation (5).

(5) $$K_{Ic}=46.22-\left(0.04909*T\right)-\left(0.8736*H\right)+\left(0.001533*T^{2}\right)+\left(0.0005344*T*H\right)+\left(0.011*H^{2}\right)+\;\left(7.486*10^{-7}*T^{3}\right)-\left(1.729*10^{-5}*T^{2}*H\right)+\left(3.099*10^{-6}*T*H^{2}\right)-\left(4.687*10^{-5}*H^{3}\right)$$

where *T*—Temperature in °C, *H*—Humidity in %. 

**Figure 11:**
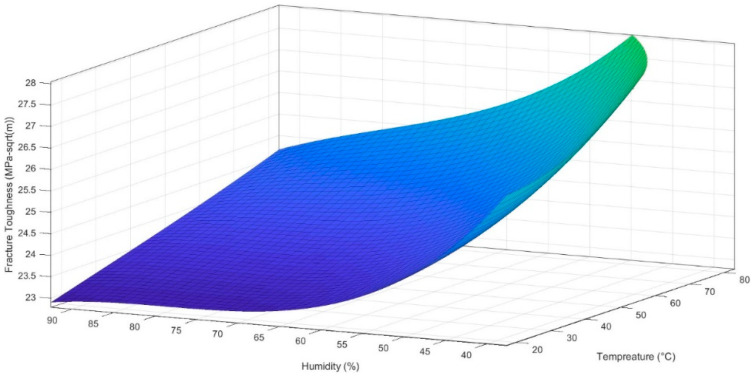
Variation of the fracture toughness for varying temperature and humidity.

The coefficient of determination of the regression model, R-square, is 0.983. Figure 11 shows the 3D surface curve obtained from the mentioned regression model.

Figure 11 shows the combined effect of temperature and humidity on the fracture toughness of the Al–Mg–Si–Mn alloy. The 3D surface plot shows that as the temperature increases, fracture toughness increases, whereas an increment in humidity decreases the material’s fracture toughness. The nonlinear cubic polynomial Equation (5) best suits the scattered points obtained from the experiment results.

### 4.2. Mapping of Curve-Fitting Graphs with Micrographs

**Figure 12:**
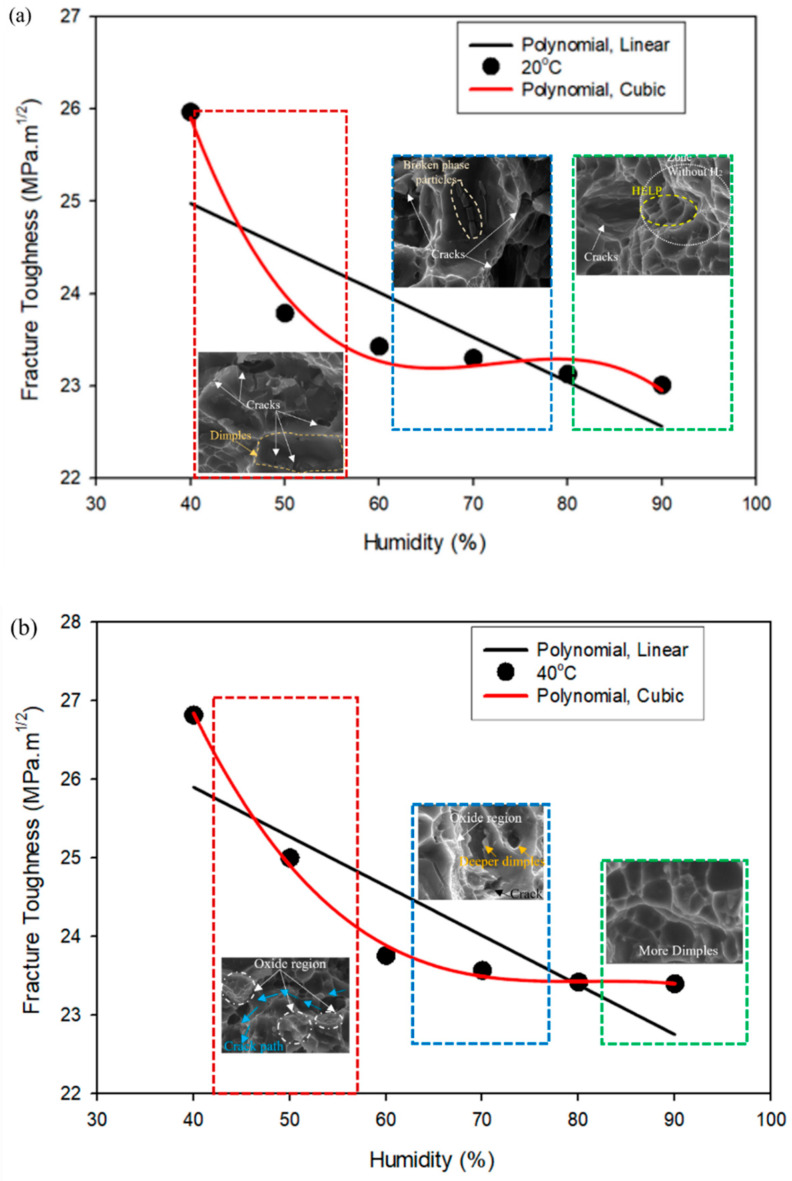

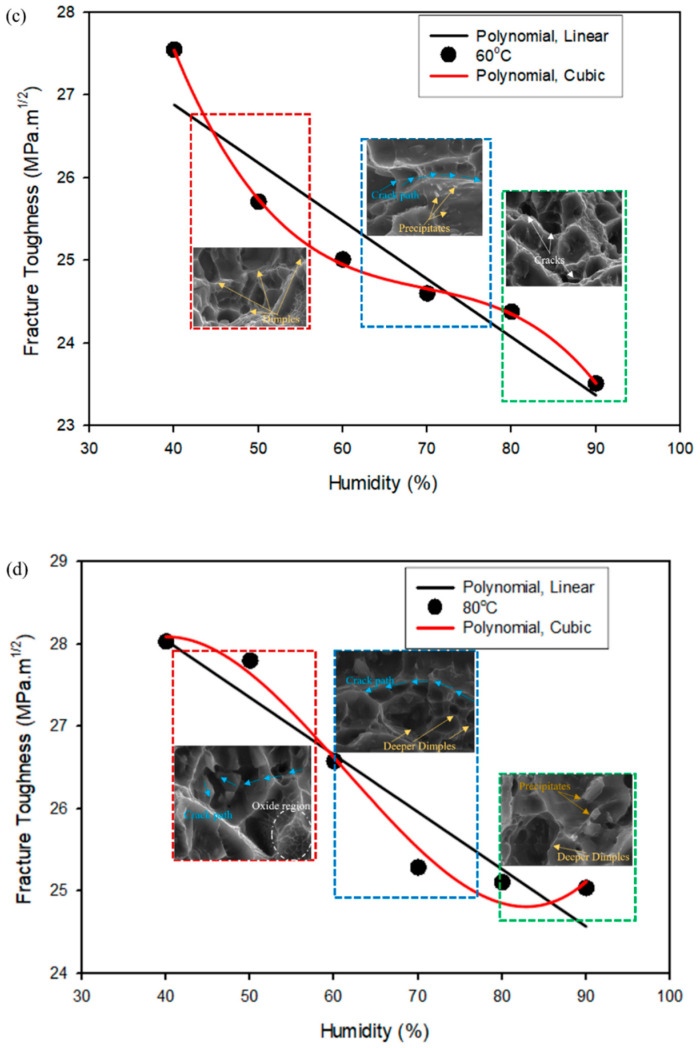
Curve fitting for different temperatures (**a**) 20 °C, (**b**) 40 °C, (**c**) 60 °C, (**d**) 80 °C vs. fracture toughness of Al–Mg–Si–Mn alloy.

**Figure 13:**
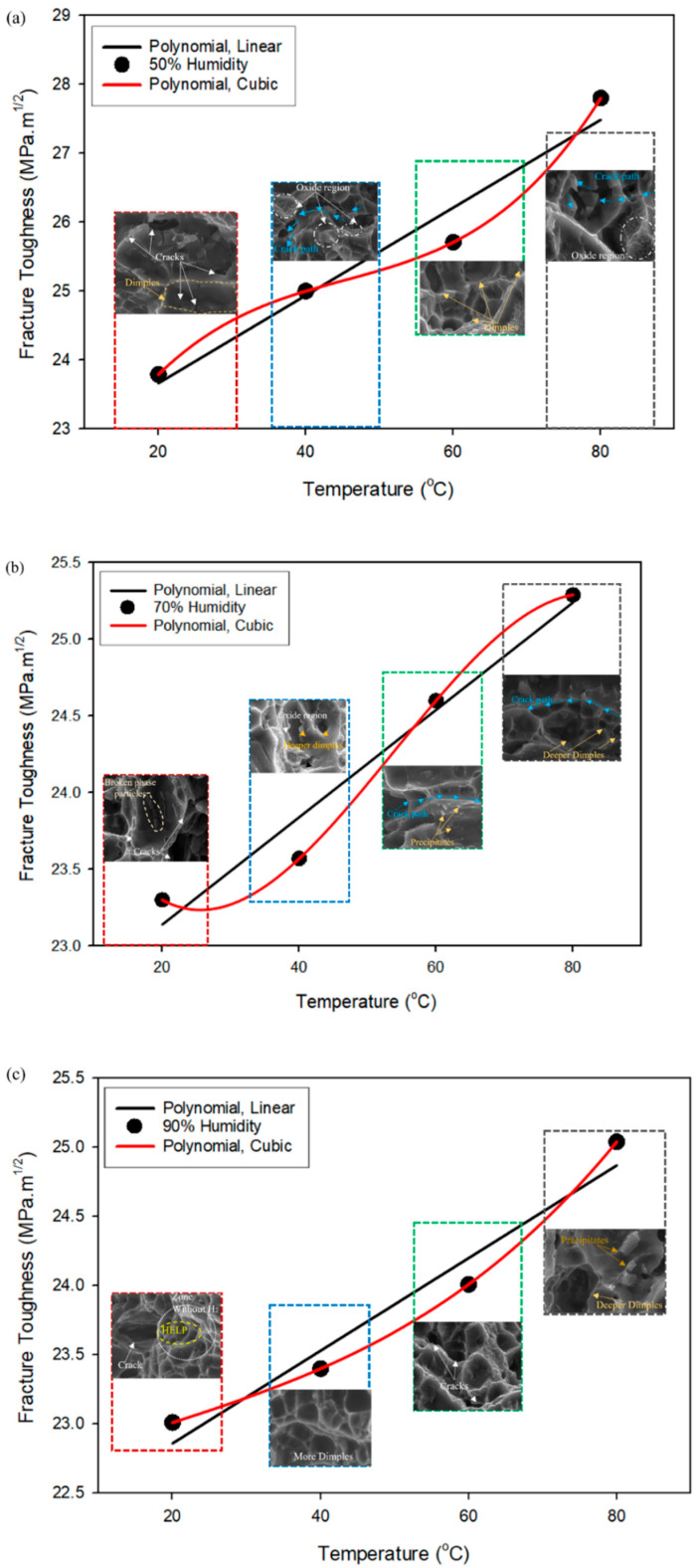
Curve fitting for different humidity (**a**) 50%, (**b**) 70%, (**c**) 90% vs. fracture toughness of Al–Mg–Si–Mn alloy.

The curve fitting for the scatter plots shown in Figure 3a,b has been carried out to find the best-fitting polynomial function. Figure 12a–d shows the temperature variations, and Figure 13a–c shows the different humidity curves fitting functions, such as linear and cubic, applied to the fracture toughness to varying temperatures and humidity conditions, respectively. 

Figure 12 and Figure 13 show that the cubic polynomial function best suits the scatter points obtained from the experiments rather than the linear polynomial function. The experiments conducted by various other researchers on the effect of humidity [28] and temperature [40] on aluminium alloy follow the same trends. Thus, it can be inferred that the effect of humidity and temperature on the mechanical properties of aluminium alloy follows the nonlinear curves. In the present work, the combined effect of a couple of loads, such as humidity and temperature, on the fracture toughness of aluminium alloy, hence, follows the nonlinear polynomial function. 

In the case of the Al–Mg–Si–Mn alloy being studied, the nonlinear variation of results is evident in the fact that cracks and corrosion on the surface are inconsistent across all environmental conditions. For example, at 50% humidity, the cracks are deeper and more pronounced. In contrast, at 70% humidity, there is evidence of broken particles, and at 90% humidity, the HELP mechanism is observed, as shown in Figure 12a. 

At 40 °C and 50% humidity, there is a crack path between two oxide layers with a significant decrement in fracture toughness. At 40 °C and 70% humidity, there are cracks in the dimples with oxide layers indicating degradation. At 40 °C and 90% humidity, there are only dimples with stable fracture toughness values, as shown in Figure 12b. 

At 60 °C and 50% humidity, the SEM images show deeper dimples and cracks between oxide layers with a significant decrement in fracture toughness, indicating a degraded state of the material. At 60 °C and 70% humidity, SEM images reveal a sign of precipitates and a crack path away from the precipitates with stable fracture toughness values. This suggests that precipitates in the material under certain environmental conditions can lead to more stable behaviour. 

At 60 °C and 90% humidity, the SEM images show only the cracks with a further reduction in fracture toughness values, indicating a high level of degradation, as shown in Figure 12c.

The mapping of SEM images using the curve fitting technique indicates that the material’s behaviour is nonlinear under different environmental conditions. At 80 °C and 50% humidity, SEM images show a crack path away from the oxide region with a little decrement in fracture toughness. At 80 °C and 70% humidity, SEM images reveal dimple cracks without an oxide layer and a significant decrement in fracture toughness. At 80 °C and 90% humidity, the SEM images show precipitation (as shown in Figure 12d) with stable fracture toughness values. These varying behaviours suggest that the material exhibits a nonlinear response to changes in environmental conditions, such as temperature and humidity. 

Figure 13 provides a visual representation of the mapping of micrographs with curve fitting for different humidity levels at various temperatures. This analysis allows for a detailed examination of the material’s mechanical behaviour under other environmental conditions. At 50% to 90% humidity, 20 °C to 80 °C, the formation of dimples, broken phase particles in dimples, cracks, and crack path indicates that the material may be undergoing plastic deformation, which can cause local stress concentrations and promote crack initiation and propagation. These observations suggest that the material’s mechanical behaviour is highly dependent on temperature and humidity, affecting its fracture toughness. The observed continuous increment in fracture toughness at 80 °C may be attributed to factors such as crack blunting due to oxide layers, crack tip shielding by plastic deformation, and crack deflection by the material’s microstructure. These factors can effectively dissipate the energy of the propagating crack, leading to an increase in fracture toughness. These factors may lead to a nonlinear increase in the material’s fracture toughness due to the formation of microcracks that can effectively dissipate the energy of the propagating crack. 

The changes in temperature and humidity influenced the tolerance of the aluminium alloy Al–Mg–Si–Mn alloy due to several interconnected scientific factors. These factors arise from the inherent properties of the material and its response to environmental conditions.

When the temperature increases, the aluminium alloy expands; when it decreases, it contracts. This expansion and contraction can lead to dimensional changes and affect the overall tolerance of the material. Changes in the temperature induce stress and strain within the material. When the aluminium alloy undergoes thermal cycling, it experiences internal stresses due to differential expansion and contraction across its structure. These stresses can affect the material’s dimensional stability, resulting in tolerance changes and nonlinear variation.

The formation of micro-voids due to increased humidity levels in the Al–Mg–Si–Mn alloy might result in hydrogen gas entrapment, leading to hydrogen embrittlement. This process is associated with reduced fracture toughness and increased crack propagation, resulting in a nonlinear relationship between humidity and fracture toughness as shown in Figure 11. 

The interaction between the aluminium alloy and the 3.5 wt. % NaCl solution can result in corrosion and oxidation. Localised thinning or material loss was experienced by the material when it underwent corrosion and oxidation. The tolerance of the material was directly impacted by these changes in dimensions, as deviations from the desired specifications occurred due to surface irregularities or thinning. In such cases, the nonlinear relationship between the corrosion-induced material changes and the resulting tolerance variations was captured by employing linear or cubic fitting functions.

The combined effect of temperature and humidity changes led to complex interactions within the aluminium alloy, resulting in nonlinear variations in dimensional stability and tolerance. The nonlinear variation arose due to the intricate relationship between temperature, humidity, and the material’s response.

### 4.3. Comparison of Experimental and Curve-Fitting Results 

In this study, an empirical model was developed (Equation (5)) to predict the mechanical behaviour of the studied material under different environmental conditions. The accuracy and reliability of the model were evaluated by comparing the model predictions with the experimental results. The experimental fracture toughness values and the calculated fracture toughness values using the empirical model (Equation (5)) are compared in Table 2. The maximum error observed between the experimental and model results was 2%.

The low error rate indicates that the empirical model successfully accurately predicted the mechanical behaviour of the studied material under different environmental conditions. This is an important finding, as it demonstrates the ability of the empirical model to capture complex microstructural changes that can influence a material’s mechanical properties. 

## 5. Conclusions

The investigation of interdependencies between the coupled effects of temperature and humidity on fracture toughness of Al–Mg–Si–Mn alloys has provided significant insights into the behaviour of these materials under real-world conditions. The following are the conclusions drawn from the investigation:

Based on the microstructure analysis conducted in this study, it was observed that the presence of phase particles at higher temperatures increased the fracture toughness of the Al–Mg–Si–Mn alloys. However, the results also indicated that higher humidity levels led to the formation and distribution of more of these particles, which resulted in a decrease in fracture toughness due to increased stress concentration. The formation of micro-voids due to increased humidity levels in the Al–Mg–Si–Mn alloys might result in hydrogen gas entrapment, leading to hydrogen embrittlement. This process is associated with a reduction in fracture toughness and an increase in crack propagation.

The results of the fracture toughness test and microstructural analysis have shown that temperature and humidity have a nonlinear, interdependent relationship that can significantly impact the alloys’ fracture toughness.

Furthermore, the developed empirical model has proven to be an effective tool for predicting the fracture toughness of Al–Mg–Si–Mn alloys under different temperature and humidity conditions. This study provides essential information to the scientific community about the interdependencies between temperature and humidity on the fracture toughness of Al–Mg–Si–Mn alloys. The model can be used to optimise the selection and design of materials in coastal environments.

## Figures and Tables

**Table 1:** Chemical composition of Al–Mg–Si–Mn alloy (wt. %) Doré Metal Services Southern Ltd.

Element	Cu	Cr	Mn	Mg	Zn	Ti	Si	Fe	Al
wt. %	0.1	0.08	0.9	1.2	0.2	0.2	1.1	0.5	Balance

**Table 2:** Comparison of experimental and empirical model results.

Sl. No.	Temperature	Humidity	Experimental *K*_*Ic*_ (MPa.m^1/2^)	Empirical Model *K*_*Ic*_ (MPa.m^1/2^) (Equation (5))	% Error
1	20	50	23.79	24.16	1.53
2	20	60	23.43	23.37	−0.26
3	20	70	23.30	23.10	−0.86
4	20	80	23.13	23.07	−0.26
5	20	90	23.01	23.01	−0.01
6	40	50	25.00	24.71	−1.14
7	40	60	23.76	23.89	0.53
8	40	70	23.57	23.60	0.12
9	40	80	23.42	23.56	0.59
10	40	90	23.40	23.50	0.44
11	60	50	25.71	25.87	0.61
12	60	60	25.00	24.87	−0.49
13	60	70	24.60	24.43	−0.73
14	60	80	24.38	24.25	−0.54
15	60	90	24.01	24.05	0.18
16	80	50	27.80	27.67	−0.46
17	80	60	26.58	26.37	−0.80
18	80	70	25.29	25.62	1.29
19	80	80	25.11	25.16	0.20
20	80	90	25.04	24.69	−1.41

## Data Availability

Data sharing does not apply to this article as no datasets were generated or analysed during the current study.
